# Do various personal hygiene habits protect us against influenza-like illness?

**DOI:** 10.1186/s12889-019-7726-9

**Published:** 2019-10-22

**Authors:** Amro K. Bin Abdulrahman, Khalid A. Bin Abdulrahman, Mansour K. Almadi, Abdulrahman M. Alharbi, Mahmoud A. Mahmoud, Mohammed S. Almasri, Tariq R. Alanazi, Rakan A. Alarifi, Abdullah A. Kilani, Omar S. Albluwi, Muaath A. Al Fraih, Yaser T. Al Otabi, Hani O. Alanazi, Waleed A. Almufarih, Abdullah M. Alokayli, Omar A. Alwhibi

**Affiliations:** Department of Public Health, College of Medicine, Imam Mohammad Ibn Saud Islamic University, Riyadh, Saudi Arabia

**Keywords:** Public health, Personal hygiene, Influenza vaccine, Influenza-like illness, Saudi Arabia

## Abstract

**Background:**

Several studies have reported an association between improvements in hand hygiene and the reductions in rates of intestinal parasitic diseases. However, only a some have addressed its link to the frequency of influenza-like illness. The current study aimed to find the correlation between personal hygiene habits and the frequency of influenza-like illness.

**Methods:**

A cross-sectional study targeting 3000 participants conducted in Riyadh city, Saudi Arabia. A systematic random sampling methodology was applied for participant from different part of Riyadh city using a computer generating system. The researcher first started by calling each participant. A full explanation was given to each participant in details (from the purpose of the research, consent to answer the questionnaire, to the explanation of the outcome definition). Each point of the questionnaire was explained to them to make sure they had excellent comprehension, and therefore, respond accurately. Descriptive statistics and Odds Ratio and its 95% confidence intervals were used to determine the association between frequency of influenza-like illness and the studied variables.

**Results:**

Two thousand eighty-two (69.4%) completed the questionnaire. The participants who spent 5–10 s in handwashing with soap and rubbing were at increased risk of more frequent influenza-like illness (odds ratio = 1.37, 1.08–1.75). Handwashing with soap and rubbing after handshaking is an independent protective habit against frequent influenza-like illness (adjusted OR = 0.59, 0.37–0.94).

**Conclusion:**

The decrease of the frequency of influenza-like illness could be done through the following: getting the influenza vaccine annually, washing hands with soap and hand rubbing not less than 15 s after getting out of the bathroom, before and after handshaking and before eating. Soap companies should invent soaps that take less rubbing time to kill bacteria, and subsequently may maximize compliance in the community.

## Background

It is thought that good hygiene habits, such as avoiding the use of other people’s personal items may help in the reduction of communicable diseases. Handwashing compliance is a recognized tool in terms of quality and care in various countries around the world [1]. Still, the infection rate is high in many parts of the world, even though hygiene education and health resources are available, so the problem arises from personal practice and behavior in the community. Hand hygiene also plays a role in minimizing acute respiratory infections [2].

The majority of influenza-like illness (ILI) is caused by many different agents that are not clinically distinguishable from one another. A variable proportion of ILI (7 to 15% on average) is caused by influenza viruses and is known as influenza [3, 4].

Seasonal influenza has been estimated to cause 90 million new acute respiratory infection cases worldwide among young children in 2008, [5] and 19,244 disability-adjusted life years lost across all age groups [6, 7].

Vaccination against influenza is the primary way to reduce the substantial health burden that seasonal influenza causes, and is the primary tool to prevent influenza infection [8].

A study during the H1N1 epidemic assessed the knowledge and attitudes of the Saudi public reported that 38.3% of participants were not convinced about the truth of published reports about this illness [9]. Of those participants, 16.1% reported receiving information from health providers about influenza-related guidelines. Another study during the same epidemic confirmed the need for prevention strategies, including immunization and improved personal hygiene in the Saudi population [10].

The hygiene habits of young people’s may be motivated by perceptions of socially acceptable behavior rather than by scientific knowledge [11]. Diseases intervention studies suggest that the risk of infectious intestinal disease can be reduced by up to 31% through hand hygiene [12]. Several studies have reported an association between improvements in hand hygiene and reductions in rates of intestinal parasitic diseases [12–17]. However, only some have addressed the link between personal hygiene, influenza vaccine, and the frequency of influenza-like illness. The current study aims to find the association between personal hygiene habits and the frequency of influenza-like illness.

## Methods

### Study design

A cross-sectional study was undertaken in Riyadh city, the capital of Saudi Arabia.

### Study population and sampling

Three thousand male and female youths and young adults aged from 16 to 45 years were selected. The authors were able to acquire their contact information through the official government Saudi Telecom database, which was clearly explained to them that the communication with them was purely for scientific purposes, and all data of their identities will remain confidential. A systematic random sampling of those randomly selected participant from different parts in Riyadh was applied using a computer generating system to make sure it is representative to the whole city.

### Study questionnaire

The researcher first started by calling each participant. A full explanation was given to each participant in details (from the purpose of the research, consent to answer the questionnaire, to the explanation of the outcome definition). Each point of the questionnaire was explained to them to make sure they had excellent comprehension, and therefore, respond accurately. Survey Monkey program was used as it had easier presentation of the questions to the participant [18]. Next, the participants were asked to answer the questionnaire, which was designed to explore the participant’s personal hygiene habits, gender, age, social status, and level of education. The study questionnaire contained 20 questions regarding habits of personal hygiene and related information and four demographic questions. The participants were asked questions that were considered the study variables, which were as follows: hand hygiene habits, use of other personal items, the action is done when sneezing, influenza vaccination and the number of times they had influenza-like illness, in which all of these were asked to be answered from the participant’s past 4 weeks to minimize possible recall bias. The questionnaire was subjected to a pilot study by 36 youth and young adult volunteers. Further improvement and rephrasing were carried out accordingly. The influenza vaccination was considered a secondary investigation of the current research.

### Exposure definitions

Different questions were asked. For example, in the past 4 weeks, do you wash your hands before eating? Always, sometimes, rarely, never. On your ordinary day, approximately how much time do you spend washing your hands? Less than 5 s, 5–10 s, 10–20 s, 20–30 s, More than 30 s. Do you wash your hands after handshaking? Always, sometimes, rarely, never. Do you wash your hands before handshaking? Do you wash your hands after getting back home? Do you wash your hands after getting out of the washroom (W.C)?

Do you use other people’s personal items? I use other people’s towels always, sometimes, rarely, never. I use other people’s used spoon. I share smoking shisha (habli babli) with others. I share the drinking straw with others. I share the same used drinking glass with others. I used to touch my eyes and nose? How many times do you get influenza-like illness in 1 year? 1–3 times/ year, 4–6 times / year, 7–10 times / year, more than 10 times / year. What do you do when you are about to sneeze? I sneeze on my hands; I sneeze on my clothes, I sneeze using napkins, I sneeze on my forearm, I sneeze on the air (empty space). Did you take the Influenza vaccine this year? Yes, no. If the answer yes in the previous question, do you take the Influenza vaccine every year? Yes, no.

### Outcome definition

The term influenza-like illness was defined based on the case definition of the World Health Organization, which is an acute respiratory infection with a measured fever of ≥38 C° and cough; with onset within the last 10 days [19].

### Data analysis

The statistical package for social science version 17.0 (SPSS Inc. Chicago, IL) was used to analyze the collected data. Descriptive statistics and Odds Ratio and its 95% confidence intervals were used to determine the association between the frequency of influenza-like illness and the studied variables. Significant associations in the crude analysis were submitted into the multivariate logistic regression model, and the Odds Ratio was reported.

## Results

Out of 3000, Saudi youth and young adults’ participants, 2082 (69.4%) completed the questionnaire. Table 1 showed that the majority were single (53.2%) and (46.8%) were married. Males were (59.8%), and the majority were at the university level (73.1%). However, the least percentage was among those who completed only elementary or intermediate levels (0.6 and 2.6%) respectively. The majority were aged 21–30 years (48.5%). The least number of the sample was aged 16–20 (14.5%).

**Table 1:** Socio-demographic characteristics of adults regarding their personal hygiene behavior in Saudi Arabia

Characteristic	Number n 2082	Percentage (%)
Age^a^		
16–20	300	14.5
21–30	1005	48.5
> 30	777	37
Gender		
Male	1246	59.8
Female	836	40.2
Marital status		
Single	1108	53.2
Married	974	46.8
Education		
Elementary	12	0.6
Intermediate	55	2.6
Secondary	493	23.7
University	1522	73.1

Data are presented as numbers and percentages from the total sample

^a^Age is in years

Table 2 showed the duration of handwashing with soap and rubbing, which were statistically significant in terms of the time spent on handwashing as it showed that the participant who spent 5–10 s of handwashing with soap and hand rubbing got influenza-like illness more frequently in comparison to those who washed their hands for long periods. Also, handwashing with soap and hand rubbing behavior was protective against influenza-like illness at all levels (rare, sometimes or always) in the following situations: before eating, after using the bathroom and after coming back home.

**Table 2:** Handwashing with soap and rubbing and frequency of having influenza-like illness among adults in Saudi Arabia

Habit	Frequency of influenza-like illness in the past 4 weeks n 2082 (%)		OR	95% Conf. Interval
	1–3^b^	> 3^b^		
Duration of hand washing				
< 5 s	144 (28.1)	356 (22.7)	REF	
5–10 s	246 (47.3)	822 (52.4)	1.37	1.08–1.75^a^
10–20 s	85 (16.6)	265 (16.9)	1.26	0.92–1.72
20–30 s	24 (4.7)	77 (4.9)	1.3	0.79–2.13
> 30 s	17 (3.3)	50 (3.2)	1.19	0.66–2.13
Hand washing before eating				
Never	270 (52.7)	965 (61.5)	REF	
Rarely	195 (38.1)	530 (33.8)	0.28	0.23–0.34^a^
Sometimes	37 (7.2)	65 (4.1)	0.49	0.32–0.75^a^
Always	10 (2.0)	10 (0.6)	0.28	0.12–0.68^a^
Hand washing after using the bathroom				
Never	386 (75.4)	1354 (86.2)	REF	
Rarely	101 (19.7)	184 (11.7)	0.52	0.4–0.68^a^
Sometimes	16 (3.1)	22 (1.4)	0.39	0.2–0.75^a^
Always	9 (1.8)	10 (0.6)	0.31	0.13–0.78^a^
Hand washing after coming back home				
Never	131 (25.6)	481 (30.6)	REF	
Rarely	165 (32.2)	489 (31.1)	0.81	0.62–1.05
Sometimes	86 (16.8)	266 (16.9)	0.84	0.62–1.15
Always	130 (25.4)	334 (21.3)	0.70	0.53–0.93^a^
Hand washing after handshaking				
Never	11 (2.1)	51 (3.2)	REF	
Rarely	100 (19.5)	383 (24.4)	0.8	0.4–1.6
Sometimes	160 (31.3)	388 (24.7)	0.5	0.26–1.03
Always	241 (47.1)	748 (47.6)	0.32	0.16–0.61^a^
Hand washing before handshaking				
Never	146 (28.5)	554 (35.3)	REF	
Rarely	135 (26.4)	399 (25.4)	0.77	0.6–1.01
Sometimes	101 (19.7)	238 (15.2)	0.62	0.46–0.83^a^
Always	130 (25.4)	379 (24.1)	0.76	0.59–1.00

*OR* Odds Ratio

The data are presented as numbers (%) and 95%Confidence Interval for comparison and Statistical significance

^a^Statistically significant difference

^b^Number of times people were affected with influenza-like illness

Statistically significant data showed that handwashing with soap and hand rubbing before and after shaking hands were also protective (Odds ratio (95% CI) = 0.62 (0.46–0.83), 0.32 (0.16–0.61) respectively.

Table 3 showed the usage of other people’s personal items with the frequency of influenza-like illness. It was found that most participants who reported using other people’s personal items all the time (e.g., towel, straw, glass, Habli Babli) had an increase in the frequency of getting influenza-like illness in percentages and odds ratio. However, it was not statistically significant.

**Table 3:** Usage of other people’s personal items and the frequency of influenza-like illness adults in Saudi Arabia

Personal Belongings	Frequency of influenza-like illness in the past 4 weeks n 2082 (%)		OR	95% Conf. Interval
	1–3**	> 3**		
Other people’s towel				
Never	11 (5.6)	26 (5.2)	REF	
Rarely	42 (21.5)	91 (18.3)	0.92	0.41–2.03
Sometimes	33 (16.9)	91 (18.3)	1.17	0.52–2.62
Always	109 (55.9)	288 (58.1)	1.21	0.53–2.34
Other people’s spoon				
Never	11 (5.6)	29 (5.8)	REF	
Rarely	34 (17.3)	97 (19.4)	0.92	0.42–2.05
Sometimes	38 (19.3)	93 (18.6)	0.86	0.50–1.48
Always	114 (57.9)	281 (56.2)	0.86	0.55–1.35
Other people’s Habli Babli (shisha)				
Never	17 (8.9)	33 (6.7)	REF	
Rarely	25 (13.0)	49 (9.9)	1	0.47–2.15
Sometimes	16 (8.3)	25 (5.0)	0.8	0.34–1.9
Always	134 (69.8)	389 (78.4)	1.5	0.81–2.77
Other people’s straw				
Never	3 (1.6)	7 (1.4)	REF	
Rarely	25 (13.0)	70 (14.1)	1.2	0.28–5.0
Sometimes	33 (17.1)	56 (11.3)	0.73	0.18–3.0
Always	132 (68.4)	364 (73.2)	1.18	0.3–4.6
Other people’s used glass				
Never	33 (16.5)	59 (11.6)	REF	
Rarely	64 (32.0)	176 (34.6)	1.54	0.92–2.57
Sometimes	44 (22.0)	108 (21.2)	1.37	0.79–2.38
Always	59 (29.5)	166 (32.6)	1.57	0.94–2.65

*OR* Odds Ratio

The data are presented as numbers (%) and 95%Confidence Interval for comparison and Statistical significance

^**^Number of times people were affected with influenza-like illness

Table 4 showed a group of miscellaneous behaviors and precautions that people may do or take with the frequency of influenza-like illness. It was found that touching eyes and nose did not affect the risk of the frequency of influenza-like illness (Odds ratio (95% CI) = 0.99 (0.76–1.3), 1.04 (0.75–1.44), 1.47 (1.0–2.15) for rarely, sometimes and always respectively). There were no statistically significant data regarding the action done when sneezing in relation to the frequency of getting influenza-like illness for covering your nose with hands, clothes, napkins or forearm, (Odds ratio (95% CI)) = 1.06 (0.8–1.4), 1.18 (0.77–1.81), 1.30 (0.98–1.73), 1.03 (0.71–1.49) respectively.

**Table 4:** Miscellaneous behaviors and influenza vaccine in relation to the frequency of influenza-like illness among adults in Saudi Arabia

Behavior	Frequency of influenza-like illness in 4 weeks n 2082 (%)		OR	95% Conf. Interval
	1–3^b^	> 3^b^		
Touch my eyes and nose				
Never	97 (18.9)	283 (18.0)	REF	
Rarely	266 (52.0)	771 (49.1)	0.99	0.76–1.3
Sometimes	98 (19.1)	298 (19.0)	1.04	0.75–1.44
Always	51 (10.0)	218 (13.9)	1.47	1.0–2.15
Action on sneezing				
On my hands	478 (30.4)	148 (28.9)	1.06	0.8–1.4
On my clothes	113 (7.2)	39 (7.6)	1.18	0.77–1.81
Using napkins	438 (27.9)	166 (32.4)	1.30	0.98–1.73
On my forearm	184 (11.7)	55 (10.7)	1.03	0.71–1.49
On the air	357 (22.7)	104 (20.3)	REF	
Influenza Vaccination this				
Year				
Yes	106 (6.8)	42 (8.2)	1.23	0.85–1.79
No	1464 (93.2)	470 (91.8)	REF	
Annual influenza vaccination				
Yes	66 (15.1)	12 (7.8)	0.47	0.25–0.90^a^
No	370 (84.9)	142 (92.2)	REF	

*OR* Odds Ratio

The data are presented as numbers (%) and 95%Confidence Interval for comparison and Statistical significance

^a^Statistically significant difference

^b^Number of times people were affected with influenza-like illness

Moreover, participants who reported taking the annual vaccination against influenza showed more protection against frequent influenza-like illness (Odds ratio (95% CI) = 0.47 (0.25–0.90).

Table 5 represents the multivariate logistic regression analysis, which showed that the annual vaccination against influenza was independently protective against frequent influenza-like illness (adjusted OR = 0.49 (0.25–0.96). Also, handwashing with soap and rubbing after handshaking was an independent protective habit against frequent influenza-like illness (adjusted odds ratio (95% CI) = 0.59 (0.37–0.94).

**Table 5:** Independent factors determining the frequency of influenza-like illness among adults in Saudi Arabia

Factor	Adjusted Odds Ratio	95% Conf. Interval.
Annual influenza vaccination	0.49	0.25–0.96^a^
Hand washing after shaking	0.59	0.37–0.94^a^

The data are presented as 95% Confidence Interval for comparison and statistical significance

^a^Statistically significant difference

## Discussion

To our knowledge, this is the first study conducted in Saudi Arabia that brings which have found a correlation between a variety of personal hygiene habits and influenza vaccine with the frequency of influenza-like illness.

This study found that handwashing with soap and rubbing was protective against influenza-like illness. Several studies were consistent with our findings [2, 20–27].

Participants spending 5–10 s in handwashing with soap and rubbing were at increased risk of more frequent influenza-like illness. Also, it turned out that handwashing with soap and rubbing before eating, after using the bathroom and after coming back home was protective from influenza-like illness regardless of the frequency. Interestingly Pires et al. have found that the time needed to kill bacteria, a person should rub for at least 15–30 s [28]. So, this makes the duration of handwashing itself a crucial factor in prevention. Most people’s underestimate the value of washing hands to maintain clean hands regularly [29].

Furthermore, washing hands with soap and rubbing before and after shaking hands were also protective against having an influenza-like illness. This finding emphasizes the importance of washing hands before and after any action — what we would call good hygiene habits.

Although Aiello et al. have found in their meta-analysis study that hand washing alone was not enough for efficacy against laboratory-confirmed influenza. However, the total percentage of respiratory diseases prevented by hand-hygiene interventions was 21% (95% CI = 5, 34%). Moreover, hand-washing education done by non-antibacterial soap was a.

precise preventative measure, as it prevented 51% (95% CI = 39, 60%) of respiratory diseases in comparison with the controlled group that did not have an intervention [27]. Nevertheless, Wong et al. have found that the overall impact of hand hygiene in protection from influenza was modest [30].

As there were previous statements from experts that using other people’s items might affect getting the influenza-like illness. However, the participants who used other people’s towel, straw, glass, and habli babli did get affected more frequently with influenza-like illness. Nevertheless, this increase was in the percentage and odds ratio, and it was not statistically significant.

It seemed like repeated touching of eyes and nose, and the action is done when sneezing (e.g., on hands, using napkins) did not affect the frequency of getting the influenza-like illness. Moreover, the annual vaccination against influenza protects against frequent influenza-like illness. There were several studies, which have supported our findings [31–33]. Annual influenza vaccines and handwashing with soap and rubbing after handshaking was independently protective against frequent influenza-like illness.

The current study supported the evidence of the link between handwashing with soap and rubbing and the decrease in the frequency of influenza-like illness. While, washing hands are considered applicable, easy to administer, and cost-effective, healthy habit that does not take a lot of time, yet protects us from many diseases. However, lack of proper hand hygiene is considered a global issue. Unfortunately, our finding leads us to think that people’s may tend to wash their hands only if there was visible dirt. Efforts regarding awareness of proper hand hygiene behavior need to be maximized.

Furthermore, the present study emphasizes the awareness of not just washing hands-on everyday occasions, such as before eating. It is recommended even on other circumstances such as after getting out of the bathroom, after coming back home, and before shaking hands. Although, the finding of this study regarding using shared items with others did not affect the frequency of getting influenza-like illness with statistical significance. However, this does not make it a healthy behavior, and therefore, caution must be taken, unless urgent situations force you to do so.

While there were previous statements regarding behaviors such as frequent touching of eyes and nose and sneezing in a wrong way, such as on hands might affect the frequency of influenza-like illness. Hence, these behaviors should not be considered significant points when preparing health promotion activities and materials. Although it is uncommon for a health provider to advise people to wash their hands with soap and to rub after shaking hands. The current study helps health providers and authorities to emphasize its importance. This study support the previous statements of experts in public health, who stated that vaccine could significantly play an essential role in the reduction of frequency of influenza-like illness.

### Limitations

The authors had to make the age group from 16 to 45 years, as in Saudi Arabia, the middle age group of 16–64 makes up the greatest share of the total population (64.8%). There was a chance of recall bias among the participants. However, the participants were asked to answer the questions based on their behavior in the last 4 weeks, which minimized the recall bias. The duration of hand-washing reported by participants might not have been accurate 100%. However, they were asked to have an approximate estimation, as it is difficult to bring all participants in one place, and observe the time they spend washing their hands. Finally, there might have been some participants who did not answer the questionnaire as they should due to un-clear comprehension of the questions. However, this matter has been addressed since the beginning, as each participant was approached individually, and was given a full explanation of each part of the questionnaire.

## Conclusion

This study presents evidence from Saudi Arabia that could contribute to the increase in public health promotion measures. The decrease of the frequency of influenza-like illness could be done through the following: getting the influenza vaccine annually, washing hands with soap and hand rubbing not less than 15 s after getting out of the bathroom, before and after handshaking and before eating. Moreover, some people don’t spend enough time performing proper hand hygiene because they are not used to it. Therefore, soap companies should invent soaps that take less rubbing time to kill bacteria, and subsequently may maximize compliance.

## Data Availability

The datasets analyzed during the current study is available from the corresponding author on reasonable request. Due to data protection restrictions and participant confidentiality, we do not make participant data publicly available.
